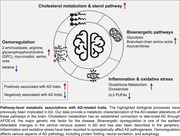# Central and Peripheral Biochemical Changes in Alzheimer's Disease: Insights from the Alzheimer Disease Metabolomics Consortium

**DOI:** 10.1002/alz.092421

**Published:** 2025-01-09

**Authors:** Richa Batra, Matthias Arnold, Xue Wang, Mariet Allen, Maria A. Wörheide, Colette Blach, Allan I. Levey, Nicholas T Seyfried, David A. Bennett, Gabi Kastenmuller, Nilüfer Ertekin‐Taner, Rima Kaddurah‐Daouk, Jan Krumsiek, Alzheimer's Gut Microbiome Project Consortium (AGMP)

**Affiliations:** ^1^ Institute for Computational Biomedicine, Englander Institute for Precision Medicine, Department of Physiology and Biophysics, Weill Cornell Medicine, New York, NY USA; ^2^ Duke University, Durham, NC USA; ^3^ Helmholtz Zentrum München, German Research Center for Environmental Health, Neuherberg Germany; ^4^ Mayo Clinic, Jacksonville, FL USA; ^5^ Helmholtz Zentrum München ‐ German Research Center for Environmental Health, Neuherberg Germany; ^6^ Emory University, Atlanta, GA USA; ^7^ Rush University, Chicago, IL USA; ^8^ Weill Cornell Medicine, New York, NY USA

## Abstract

**Background:**

Our Alzheimer Disease Metabolomics Consortium (ADMC), part of the Accelerating Medicines Partnership for AD (AMP‐AD) and in partnership with AD Neuroimaging Initiative (ADNI), applied state‐of‐the‐art metabolomics and lipidomics technologies combined with genomic and imaging data to map metabolic failures across the trajectory of the disease. Our studies confirmed that peripheral metabolic changes influenced by the exposome inform about cognitive changes, brain imaging changes, and ATN markers for disease confirming that peripheral and central changes are connected, in part through the metabolome.

**Methods:**

To map the biochemical changes in AD, we used various targeted and untargeted metabolic platforms to profile ∼800 postmortem brain tissue, and ∼ 5000 blood samples.

**Results:**

Recently, we built a comprehensive reference map of extensive AD‐related metabolic changes in brain, spanning multiple AD‐related traits, including neuropathological b‐amyloid and tau tangle burden, as well as late‐life cognitive performance. Using this resource, we extracted novel metabolic including bioenergetic pathways, cholesterol metabolism, neuroinflammation, broad impairment of osmoregulation, an imbalance between excitatory/inhibitory neurotransmitter ratios and identification of tau load as a potential driver of metabolic dysfunction in the AD brain, with minimal contributions from b‐amyloid load. As AD and progressive supranuclear palsy (PSP) share the pathological feature of tauopathy and metabolic alterations, we compared their metabolomic profiles to identify shared biological pathways that could be targeted for therapeutic interventions. Our findings indicate that both diseases display oxidative stress, mitochondrial dysfunction, and tau‐induced polyamine stress response.

**Conclusion:**

Overall, through our studies, (1) We identified biochemical processes altered in AD, with findings supported across both metabolomic and proteomic data, indicating multimodal deregulation. (2) Our research pinpointed widespread AD‐related biochemical changes across various brain regions with differing levels of neuropathology. While there are many overlapping changes across the brain regions, each region also has its distinct metabolic alterations. (3) We identified biochemical processes disrupted by AD, with parallel findings in other neurodegenerative diseases, hinting at broader implications in neurodegenerative research. Currently, we are working on mapping widespread connections of the brain metabolome with various determinants of AD namely genome, gut microbiome, exposome, and linking with peripheral metabolic alterations in AD.